# Long-term monitoring of woody plants of Doñana shrublands (SW Spain) from 2008 to 2023

**DOI:** 10.3897/BDJ.12.e139074

**Published:** 2024-12-11

**Authors:** Ricardo Díaz-Delgado, Mizar Torrijo-Salesa, Luis Alfonso Ramírez González, Antonio Alcaide, David Antonio Paz Sánchez, David Aragonés, Diego López, Isidro Román Maudo, José Ruiz-Martín, Javier Bustamante, Rocío Márquez-Ferrando

**Affiliations:** 1 Estación Biológica de Doñana-CSIC, Sevilla, Spain Estación Biológica de Doñana-CSIC Sevilla Spain; 2 ICTS-Reserva Biológica de Doñana, Almonte, Spain ICTS-Reserva Biológica de Doñana Almonte Spain

**Keywords:** Doñana Biological Reserve, line-intercept method, long-term ecological research, percentage cover, sampling event, species coverage, terrestrial vegetation, Unique Scientific and Technical Infrastructure

## Abstract

**Background:**

The long-term monitoring of the plant cover of Doñana shrublands is part of a harmonised protocol for the Long-term Ecological Monitoring Programme of Natural Resources and Processes targeting Terrestrial Vegetation. The general aim of this protocol is to monitor and assess the dynamics and trends of shrubland plant communities in Doñana. For shrublands, percentage cover is recorded annually, starting in 2008, by the Doñana Long-Term Monitoring Team in one field sampling campaign per year during the flowering season (between March and May) across 21 permanent square plots (15 m x 15 m). Permanent plots were located according to stratified random sampling according to the topographic gradient defining the main shrubland species dominance in the Doñana Biological Reserve. Cover is measured using the line intercept method in three transects inside the plots of 15 m length, orientated from west to east and located at fixed points of 2.5, 7.5 and 12.5 metres on both sides of the plot. Using the line-intercept method, the coverage of each species per individual is measured with a measuring tape, recording its class age (adult or seedling) and canopy status (green or dry) as a living or dead specimen. The average plant height is recorded for every transect. This method enables the calculation of the total percentage cover per species and plant density for transects and plots, as well as the total percentage cover per class age and the total percentage cover of dry and green canopies and bare soil. The annual species richness and diversity of woody plants can also be calculated for every plot.

**New information:**

This paper presents the latest published version of the standardised dataset of percent cover per woody plant species of Doñana long-term monitoring plots available at GBIF.org.

## Introduction

Shrublands are widely distributed plant communities in the Mediterranean Basin. Shrubs are small-to-medium-sized perennial woody plants occupying vast areas in the Mediterranean Region. Shrubland plant communities provide several ecosystem services (Riera et al. 2007). Shrub communities have been reduced in the last decades due to changes in land uses, wildfires, aridification and global warming. Many research studies have focused on shrublands as a relevant indicator of climate and global changes (Gallego Fernández et al. 2004, Riera et al. 2007, Lloret et al. 2016, Pérez-Ramos et al. 2017, de la Riva et al. 2017). The long-term monitoring and research of these communities provides valuable information to assess spatial and temporal dynamics and understand the effects of severe disturbances (Pérez-Ramos et al. 2017).

Mediterranean-type shrubs widely dominate the vegetation in the terrestrial aeolian sands of Doñana National Park. A xerophytic shrub community dominates the drier and stabilised sand dunes, mainly composed of Cistaceae and Lamiaceae species (*Halimiumhalimifolium* (L.) Willk., *H.commutatum* Pau, *Cistuslibanotis* L., *Lavandulastoechas* L. and *Rosmarinusofficinalis* L.). Low areas and depressions are usually closer to the water table and are dominated by heathland (*Ericascoparia* L., *Callunavulgaris* (L.) Hill) protected by the European Habitats Directive as Atlantic decalcified fixed dunes (Calluno-Ulicetea, Habitat type 2150). A transitional plant community has also been described between xerophytic shrubs and heathland as a mixed scrub of species from humid and xeric shrublands. Mediterranean shrublands cover a large area of Doñana National Park (ca. 7000 ha in 2019, around 33% of terrestrial vegetation according to ICTS-Doñana (2020)). Several factors, such as distance to water table or grazing intensity (Muñoz-Reinoso 2009, Jiménez and Díaz-Delgado 2017) are involved in the spatial distribution so that three main types of shrubland communities can be found in Doñana: xerophytic "white" shrubs, higrophytic "dark" shrubs, and transitional shrubland (Muñoz-Reinoso 2009). Doñana's shrublands have a high susceptibility to climate change due to their extreme ecological position, coping with extreme droughts, erosion, overgrazing, pests, wildfires and human impacts, such as conservation management for rabbits and Iberian Lynx, lowering of the water table etc. Many of these plant communities are listed under the EU Habitats Directive and play a very relevant ecological role in stabilising sand dunes and providing shelter and food to the fauna of Doñana protected area. Long-term ecological research of Doñana shrublands, based on a landscape-scale approach, is providing very relevant insights on woody plants diversity, dynamics, and resilience of this emblematic ecosystem.

## Project description

### Title

Long-term Doñana monitoring by the Unique Scientific and Technical Infrastructure of Doñana Biological Reserve (ICTS-RBD) (ref.: 202030E286)

### Personnel

Ricardo Díaz-Delgado, Mizar Torrijo-Salesa, Luis Alfonso Ramírez González, Antonio Alcaide, David Antonio Paz Sánchez, David Aragonés, Diego López, Olga Ceballos, Isidro Román Maudo, Alejandria Rojas, Juan Tenorio, Katrin Schmidt, Jose Ruíz-Martín, Javier Bustamante, Rocío Marquez-Ferrando

### Study area description

Doñana LTSER (Long-Term Socio-Ecological Research) Platform. Doñana Protected Area. Doñana National Park. Doñana Biological Reserve (RBD).

### Design description

The Doñana Long-Term Monitoring Programme has been carried out by ICTS-RBD (Unique Scientific and Technical Infrastructure of Doñana Biological Reserve) since 2004. Certain monitoring and survey activities have already started in the 1980s, focusing on birds and endangered species, such as the Iberian Lynx or the Imperial Eagle. The integrated programme started in 2003, when it was extended and funded to monitor biodiversity and ecological processes targeting species, habitats and populations, as well as ecosystem structure, function and services. Long-term data systematically collected provide a baseline for decision-making and the assessment of management actions in order to minimise the impact of global change and local drivers. Results and reports are annually published and provided to the Protected Area Managers and Practitioners and to the regional authorities through the CSIC open access repository.

### Funding

National Parks Autonomous Agency (OAPN) between 2002–2007; Singular Scientific and Technical Infrastructures from the Spanish Science and Innovation Ministry (ICTS-MICINN); Ministry of Agriculture, Livestock, Fisheries and Sustainable Development from the Regional Government of Andalusia (CAGPDES-JA) since 2007; and Doñana Biological Station from the Spanish National Research Council (EBD-CSIC) provide in-kind and direct funding to maintain the programme. Finally, the project has also benefitted from the eLTER Plus INFRAIA Research Project (Horizon 2020 EU Programme, Agreement No. 871128), the eLTER H2020 INFRAIA project (Horizon 2020 EU Programme, Agreement No. 654359) and the SUMHAL Research Project funded by FEDER actions [SUMHAL, LIFEWATCH-2019-09-CSIC-13, POPE 2014-2020] from the Ministerio de Ciencia, Innovación y Universidades.

## Sampling methods

### Study extent

The study area is located inside Doñana Protected Area in southwest Spain, where permanent plots are spread across Doñana Biological Reserve (60 km^2^). The climate is Mediterranean sub-humid with Atlantic coast influence, resulting in wet mild winters and dry warm summers. The rainy season occurs between October and April, with a peak in December–January (average rainfall is about 550 mm). Doñana's four main ecosystems are monitored, including: temporary marshes, active sand dunes, Mediterranean shrublands and woodlands and Doñana's shoreline of 30 km length. Under the vegetation topic, shrubland plant communities are monitored in 21 permanent plots sampled once per year during the peak flowering season, between March and May.

### Sampling description

**Figure 1.**
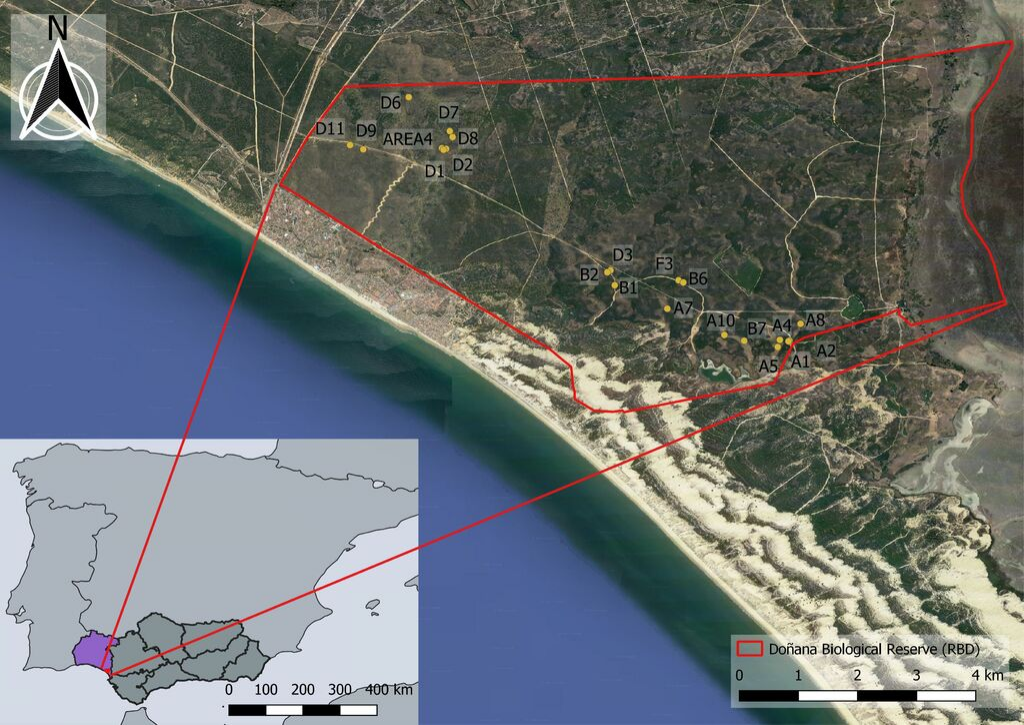
Geographic location of the 21 permanent plots (with their identification codes) monitored and available in the dataset. All plots are located inside the Doñana Biological Reserve, RBD (red polygon). Doñana protected area is located in the south-western part of the Iberian Peninsula.

**Figure 2.**
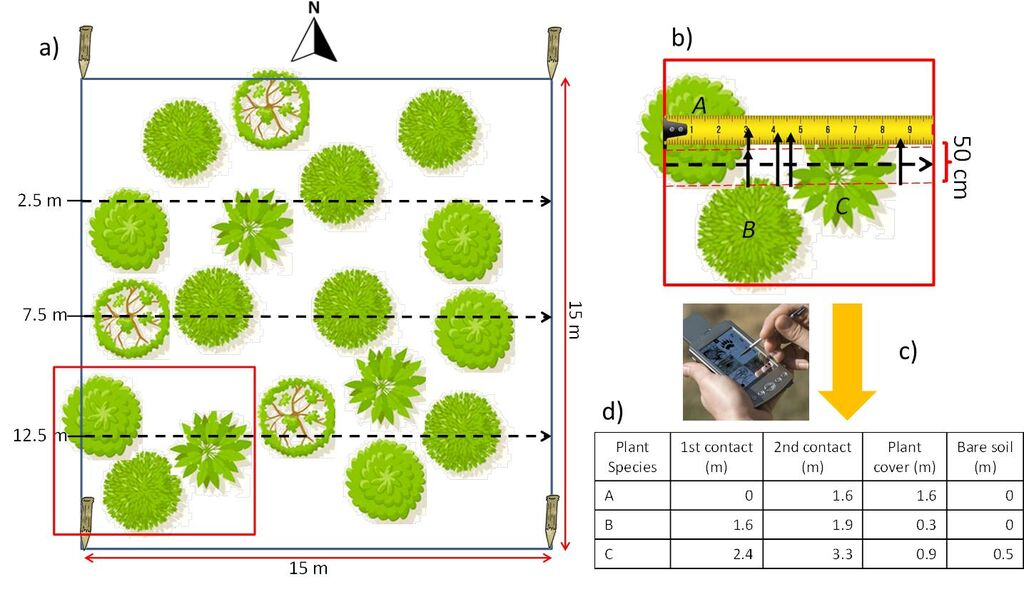
Graphical scheme of the sampling procedure. a) Zenithal view of one monitoring plot with the different woody plant canopies and transects located inside the plot; b) zoom in the red square in figure a) showing the interceptions of every canopy with the measurement tape and the strip band considered for interceptions; c) field data collection with a digital device; d) resulting table of the example in b).

The long-term monitoring of Doñana shrublands started in 2008 by setting 21 permanent plots across the Doñana Biological Reserve. Each plot is sampled during one sampling campaign per year along the flowering season (between March and May). A total of 21 permanent square plots (15 m x 15 m) were located according to stratified random sampling (Fig. 1). Stratification was based on the three main types of shrub communities found on the stabilised sand dunes according to water table depth in summer: xerophytic white shrub > 4 m; transitions shrub > 1 m and < 4 m; hygrophytic dark shrub < 1 m. Woody plant cover is measured using the line intercept method across three transects per plot of 15 m length, orientated from west to east and located at fixed points of 2.5, 7.5 and 12.5 metres on both sides of the plot. Using the line-intercept method, the contacts of each plant individual are recorded in a band strip of 50 cm along the measuring tape (Cummings and Smith 2000), together with the plant species, the class age, either adult (size class > 25 cm) or seedling (size class < 25 cm), the canopy status as a living or dead specimen and the average canopy height per transect, visually estimated (Fig. 2). According to the plant structure of the monitored woody plant communites, only one vertical stratum, the dominant and taller, is measured so that plants or seedlings in the understorey are disregarded, being those very infrequent. The method enables the calculation of the percentage cover for each species across every transect and for the whole plot (as the summatory of the three transects), including total data per species on class age and percentage of dry and green canopies, additionally to the percentage cover of bare soil, plant species density, species richness and plant species diversity. The maximum sampling time per plot was 75 minutes, being 40 minutes the average time, including stakes search and plot deployment. This permanent sampling is used as ground-truthing for further landscape monitoring using different remote sensing data sources (satellite, airborne and unmanned Aerial vehicle (UAV)), enabling the validation of shrubland mapping at larger scales (Jiménez and Díaz-Delgado 2015, Jiménez and Díaz-Delgado 2017). The yearly collected data on species occurrence and abundance in the monitored plots are used to train classifiers and produce maps of spatial distribution of main shrubland species in the Doñana Biological Reserve.

### Quality control

Taxonomic identification is assessed by different observers at the time in the field, using flowers and fruits to complement the correct identification. Plots are located with permanent stakes and coordinates collected with a D-GPS (ca. < 1 m horizontal accuracy). Although more than 10 observers have participated in the sampling, 80% of the sampling was led by the same observer. Data were digitally collected using mobile devices by means of a specifically designed Cybertracker sequence. This procedure guides the observer through a sequence of screens in a step-by-step way, some of them mandatory to prevent the loss of data. A map and a list of the plots, as well as a plant species list and observer names, are also available in the sequence for the observer. Unidentified plants in the field were later taxonomically identified. Contiguous individuals from the same species were recorded separately to improve plant density calculations. Data were transferred to a central Cybertracker database used for basic quality assessment, where the most frequent error (95%) corresponds to wrong tape measurements, which were corrected according to the previous record. Interannual plot percentage cover comparisons were also used to assess consistency in plant species occurrences and relative abundance, although the plant cover dynamics of these plant communities are highly variable.

### Step description

Along each plot, there are three different transects of 15 metres, on which a measuring tape is extended. The three transects were distributed at fixed distances from the western side of the plot, the first one being 2.5 metres from the NE corner, the second one is 7.5 metres and the last one is 12.5 metres (Fig. 2). All the individuals intercepted by measuring tape were identified and the tape distance of the initial and final contact points was recorded in order to calculate the total cover in the transect per species. Ancillary data on age class (adult or seedling: plant height under 25 cm) and canopy status (green or dry), which means the vitality of a living or dead specimen, was also recorded for each individual. The average plant height was recorded for every transect. The percentage green and dead cover of each species was calculated per transect by adding up the measured interceptions per canopy as shown in Fig. 2d and finally provided in percentage of the total measured distance for all the three transects (45 m). Data were collected in the field using a CyberTracker-programmed sequence and downloaded as Excel or CSV-files.

## Geographic coverage

### Description

The 21 shrubland permanent plots included in this long-term monitoring were set across the Doñana Biological Reserve (RBD) (red line in Fig. 1). These plots are included in the Doñana LTSER Platform, which contains the Doñana Protected Area.

### Coordinates

36.983 and 37.031 Latitude; -6.548 and -6.463 Longitude.

## Taxonomic coverage

### Description

For the whole monitoring period, technicians have identified 34 different species, eight generic identifications (i.e. genus) and a few individuals remain indeterminate. Taxa included three classes, 11 orders and 16 different families of terrestrial plants (Table 1). The most abundant families are Cistaceae, Lamiaceae, Fabaceae and Ericaceae, making up 93% of the total percentage cover (including all years and transects) (Table 1).

### Taxa included

**Table:** 

Rank	Scientific Name	
kingdom	Plantae	
phylum	Tracheophyta	
class	Liliopsida	
order	Asparagales	
family	Asparagaceae	
family	Asphodelaceae	
order	Poales	
family	Cyperaceae	
class	Magnoliopsida	
order	Asterales	
family	Asteraceae	
order	Caryophyllales	
family	Caryophyllaceae	
family	Plumbaginaceae	
order	Ericales	
family	Ericaceae	
order	Fabales	
family	Fabaceae	
order	Lamiales	
family	Lamiaceae	
family	Oleaceae	
order	Malvales	
family	Cistaceae	
family	Thymelaeceae	
order	Myrtales	
family	Myrtaceae	
order	Santalales	
family	Santalaceae	
class	Pinopsida	
order	Pinales	
family	Cupressaceae	
family	Pinaceae	

## Traits coverage

### Plant traits

**Figure 3.**
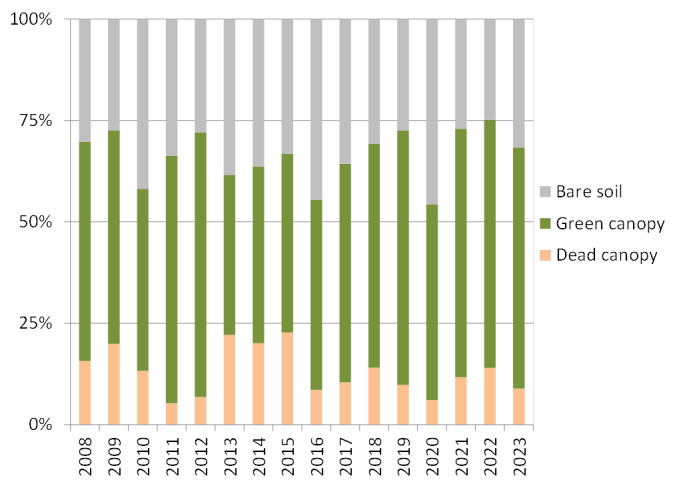
Yearly total percentage cover of woody plant species according to canopy status (green/living canopies and dry/dead canopies) and bare soil.

**Figure 4.**
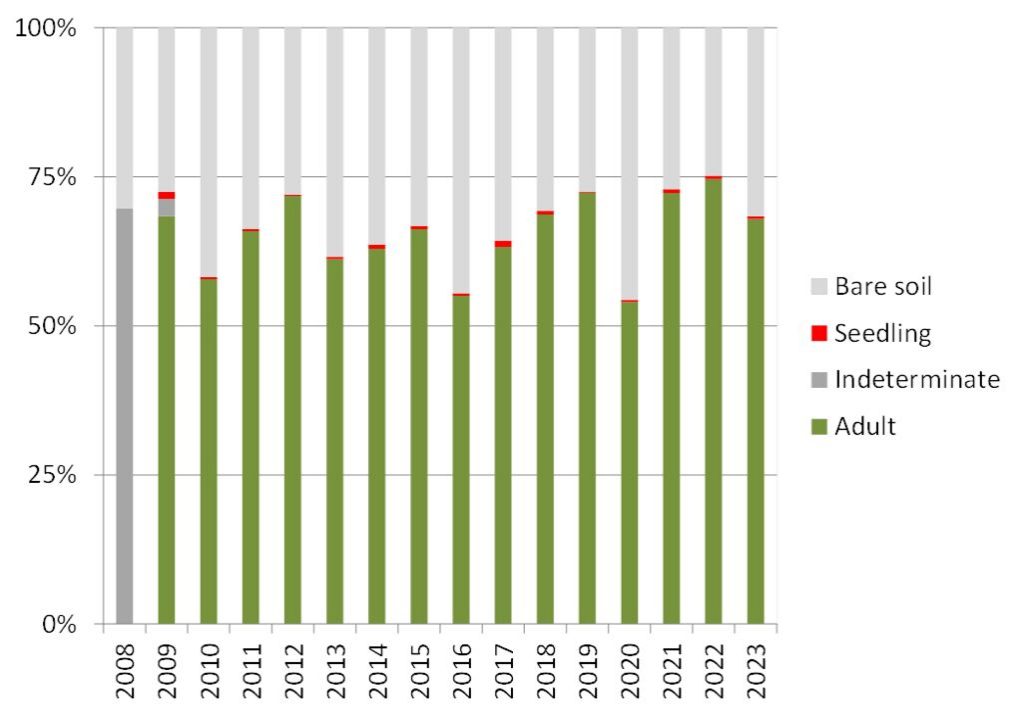
Yearly total percentage cover of woody plant species according to age class (adult or seedling) and percentage cover of bare soil. Age class was not recorded in the 2008 sampling event.

With the line-intercept method, the linear coverage intercepted with the measuring tape was accounted, i.e. the initial and final contact of each individual. From this collected raw data, we calculated plant percentage cover for every plant species as the sum of the linear distance covered by each species per transect divided by the total length of the transect (15 m). Additionally, plant canopy status (living or dead canopy; Fig. 3) and age class (adult or seddling: < 25 cm; Fig. 4) for every individual were recorded, as well as the estimated average plant height per transect. These traits enhanced the study of shrubland dynamics at plot (Figs 3, 4) and transect scales.

## Temporal coverage

### Notes

From 2008-04-08 to 2023-05-11. Dataset will be updated every 5 years.

## Usage licence

### Usage licence

Other

### IP rights notes

This work is licensed under a Creative Commons Attribution (CC-BY 4.0) Licence.

## Data resources

### Data package title

Long-term monitoring of woody plants of Doñana shrublands 2008-2023

### Resource link

https://www.gbif.org/dataset/deca479d-0832-4e4b-8c94-a09f32a80adb

### Alternative identifiers

https://doi.org/10.15470/io6caz

### Number of data sets

1

### Data set 1.

#### Data set name

Long-term monitoring of woody plants of Doñana shrublands 2008-2023

#### Data format

Darwin Core

#### Description

The dataset by Díaz-Delgado et al. (2024) contains three interconnected tables in text files: sampling events (Event core), occurrences (Occurrence extension) and extended Measurement or Fact extension (MoF) for the yearly percentage cover of woody plant species of Doñana shrublands from 2008 to 2023 measured along three permanent transects, located in 21 permanent square plots (15 m x 15 m). The dataset also includes class age (adult or seedling) and plant canopy status (living or dead specimen).

**Data set 1.** 

Column label	Column description
id (Event core, Occurrence extension, MoF)	Identifier of the the sampling event.
type (Event core)	The nature of a record.
licence (Event core)	Licence of dataset.
institutionID (Event core)	An identifier for the institution having custody of the information referred to in the record.
datasetID (Event core)	Identifier of the dataset including DOI.
institutionCode (Event core)	The name (or acronym) in use by the institution having custody of the object(s) or information referred to in the record.
datasetName (Event core)	Name of the published dataset.
eventID (Event core, Occurrence extension, MoF)	An identifier for the set of information associated with a dwc:Event.
parentEventID	An identifier for the broader dwc:Event that groups this and potentially other dwc:Events.
samplingProtocol (Event core)	The references to the protocol used for the event.
sampleSizeValue (Event core)	The numeric value for a measurement of the size of the sample in an event (length of the transect or the area of a plot).
sampleSizeUnit (Event core)	The unit of measurement of the size of the sample in an event.
samplingEffort (Event core)	The amount of effort in minutes expended during the event.
eventDate (Event core)	The date during which the event occurred.
eventTime (Event core)	The time during which the event occurred.
year (Event core)	The year during which the event occurred.
month (Event core)	The month during which the event occurred.
day (Event core)	The day during which the event occurred.
habitat (Event core)	A category or description of the habitat in which the eventID occurred.
locationID (Event core)	An identifier for the Location information.
continent (Event core)	The name of the continent in which the location occurs.
country (Event core)	The name of the country in which the location occurs.
countryCode (Event core)	The standard code for the country in which the location occurs.
stateProvince (Event core)	The name of the province in which the location occurs.
county (Event core)	The name of the county in which the location occurs.
municipality (Event core)	The name of the municipality in which the location occurs.
locality (Event core)	The specific description of the transect in the plot.
minimumElevationInMetres (Event core)	The lower altitude above sea level in metres.
maximumElevationInMetres (Event core)	The higher altitude above sea level in metres.
verbatimElevation (Event core)	The original altitude above sea level of the Location.
locationRemarks (Event core)	Comments or notes about the location.
decimalLatitude (Event core)	The geographic latitude (in decimal degrees) of the geographic centre of the sampling plot.
decimalLongitude (Event core)	The geographic longitude (in decimal degrees) of the geographic centre of the sampling plot.
geodeticDatum (Event core)	The geodetic datum upon which the geographic coordinates given in decimalLatitude and decimalLongitude are based.
coordinateUncertaintyInMetres (Event core)	The horizontal distance (in metres) from the given dwc:decimalLatitude and dwc:decimalLongitude describing the smallest circle containing the whole of the dcterms:Location.
modified (Occurrence extension)	Date of modification.
language (Ocurrence extension)	Language of dataset.
collectionCode (Occurrence extension)	Code of the monitoring collection.
basisOfRecord (Occurrence extension)	Method of species identification.
dynamicProperties (Occurrence extension)	Additional measurements, facts, characteristics or assertions about the record. In this case, the vitality, an indication of whether a plant was alive or dead at the time of observation.
occurrenceID (Occurrence extension)	Identifier for the occurrence.
recordedBy (Occurrence extension)	Names of observers responsible for recording the original occurrence.
organismQuantity (Occurrence extension)	Value of the species percent cover per transect.
organismQuantityType (Occurrence extension)	Type of measurement per occurrence (percentage cover).
lifeStage (Occurrence extension)	Age class of the plant species (adult/seedling).
identifiedBy (Occurrence extension)	Name or names of the Observer/s identifying the taxon.
scientificName (Occurrence extension)	Species scientific name.
kingdom (Occurrence extension)	Kingdom of the species.
phylum (Occurrence extension)	Taxonomic Phylum of the species.
class (Occurrence extension)	Taxonomic Class of the species.
order (Occurrence extension)	Taxonomic Order of the species.
family (Occurrence extension)	Taxonomic Family of the species.
genus (Occurrence extension)	Taxonomic Genus of the species.
specificEpithet (Occurrence extension)	Taxonomic Epihet of the species.
taxonRank (Occurrence extension)	Taxonomic Rank of the identification.
scientificNameAuthorship (Occurrence extension)	Authorship of scientific name.
measurementID (MoF)	Identifier for the measurementOrFact.
measurementType (MoF)	The nature of the measurement.
measurementValue (MoF)	The value of the measurement.
measurementUnit (MoF)	The unit of the measurement value.
measurementDeterminedDate (MoF)	The date on which the measurement was made.
measurementDeterminedBy (MoF)	Names of observers who determined the value of the measurement.
measurementMethod (MoF)	Description of the method used to determine the measurement.

## Figures and Tables

**Table 1.** Taxa included in the dataset (class, order and family). The percentage cover for every family is calculated as the total plant cover from all sampling events.

Class	Order	Family	Representation (%)
Liliopsida	Asparagales	Asparagaceae	0.145
		Asphodelaceae	0.009
	Poales	Cyperaceae	0.003
Magnoliopsida	Asterales	Asteraceae	1.428
	Caryophyllales	Caryophyllaceae	0.004
		Plumbaginaceae	1.428
	Ericales	Ericaceae	9.057
	Fabales	Fabaceae	18.456
	Lamiales	Lamiaceae	28.246
		Oleaceae	0.479
	Malvales	Cistaceae	37.311
		Thymelaeaceae	0.029
	Myrtales	Myrtaceae	0.004
	Santalales	Santalaceae	0.073
Pinopsida	Pinales	Cupressaceae	0.943
		Pinaceae	1.911
Indeterminate		Indeterminate	0.472
